# Impact of administration route and gene polymorphisms on the serum concentration of voriconazole among Chinese patients with hematologic malignancies

**DOI:** 10.3389/fphar.2025.1445583

**Published:** 2025-07-17

**Authors:** Zhiyao Chen, Menghua Zhang, Lin Wang, Yuzhu Cheng, Liyan Miao

**Affiliations:** ^1^ Department of Pharmacy, The First Affiliated Hospital of Soochow University, Suzhou, Jiangsu, China; ^2^ School of Pharmacy, Suzhou Vocational Health College, Suzhou, Jiangsu, China

**Keywords:** voriconazole, serum concentration, hematologic malignancies, gene polymorphisms, CYP3A4, CYP2C19, ABCB1

## Abstract

**Aims:**

Voriconazole (VRC) is recommended as the first-line treatment for invasive fungal diseases (IFDs). Therapeutic drug monitoring (TDM)-based dose adjustments can be performed to implement the individualized use of VRC in clinical practice. Numerous studies have shown significant interindividual differences in serum VRC concentrations. It is important to identify risk factors for variations in VRC concentrations to develop TDM-based individualized VRC therapy. However, few studies have examined the impact of drug administration routes on VRC concentrations or the impact of gene polymorphisms on VRC concentrations under different administration routes in Chinese patients. This study aimed to investigate the effects of different administration routes and gene polymorphisms of *CYP2C19*, *CYP3A4* and *ABCB1* on serum VRC concentrations among Chinese patients with invasive aspergillosis.

**Methods:**

Patients (n = 160) who were administered VRC for the prophylaxis/treatment of IFDs were enrolled in this study. Quantitative analysis of VRC was performed via high-performance liquid chromatography coupled with tandem mass spectrometry. Nine types of single-nucleotide polymorphisms (SNPs) within *CYP2C19*, *CYP3A4* and *ABCB1* were detected via multiplex PCR and next-generation sequencing.

**Results:**

The C_min_ of intravenous VRC was greater than the C_min_ of oral VRC (2.3 vs. 1.5 μg/mL, respectively, *P* = 0.0006). The C_min_ of serum VRC appears to be greater in those taking VRC by Q12h than in those taking Bid and Qd when administered orally (3.8 vs. 1.4 μg/mL, respectively, *P* = 0.0045; 3.8 vs. 0.8 μg/mL, *P* = 0.0173). Within the IV + Oral and Oral groups of *CYP2C19*, the C_min_ of the serum VRC in the NMs was significantly lower than that in the IMs (1.42 vs. 2.21, *P* = 0.0108; 1.03 vs. 1.89, *P* = 0.0386). Within the IV group of *CYP3A4* rs4646437, the C_min_ of the serum VRC in the GGs was significantly greater than that in the GA + AA group (2.41 vs. 1.43, respectively, *P* = 0.0402). Similarly, in both the IV + Oral and IV groups of *CYP3A4* rs2242480, the C_min_ of serum VRC in the CCs was markedly greater than that in the (CT + TT)s (2.18 vs. 1.47, respectively, *P* = 0.0292; 2.47 vs. 1.45, respectively, *P* = 0.0173). Moreover, among the oral groups of patients with *ABCB1* rs1128503, patients with the wild-type genotype presented significantly greater serum VRC C_min_ than those with the mutant genotype (1.89 vs. 1.13, respectively, *P* = 0.0477).

**Conclusion:**

The C_min_ of intravenous VRC was greater than the C_min_ of oral VRC when patients were treated with the recommended dosage. Oral administration of VRC via Q12h is optimal for obtaining a higher C_min_ of serum VRC. Furthermore, attention should be given to VRC serum concentrations in patients with mutations in *CYP2C19*. The *CYP3A4* rs2242480 and *CYP3A4* rs4646437 genotypes may primarily affect VRC concentrations during intravenous administration, whereas *ABCB1* rs1128503 primarily affects VRC concentrations during oral administration.

## 1 Introduction

Invasive fungal diseases (IFDs) are the most common causes of infection-related morbidity and mortality in patients with hematologic malignancies (Bassetti et al., 2021; Stemler et al., 2023; Godoy et al., 2022). Voriconazole (VRC) is a broad-spectrum triazole antifungal agent and is recommended as the first-line treatment for IFDs (Malani et al., 2015). Numerous studies have reported a relationship between VRC concentrations and clinical efficacy and toxicity (Boglione-Kerrien et al., 2023; Hoenigl et al., 2013). However, VRC has nonlinear pharmacokinetics, and considerable inter- and intraindividual variability in VRC serum concentrations have been observed in various patients who received equal doses (Zhong et al., 2018; Theuretzbacher et al., 2006). Although therapeutic drug monitoring (TDM)-based dose adjustments can be performed to optimize VRC concentrations, the first dose is an important factor that influences subsequent treatment. Therefore, knowledge of potential factors that contribute to variations in VRC concentrations is critical for developing individualized VRC therapy.

Intravenous administration facilitates rapid drug entry into the circulatory system, thus enabling expedited dissemination throughout the bloodstream to target sites of infection with heightened drug concentrations. Furthermore, intravenous administration can ensure swift and potent drug delivery to combat infections, particularly those that benefit critically ill patients or those requiring urgent infection control measures (Waitt et al., 2004). On the other hand, oral administration necessitates absorption through the gastrointestinal tract, subjecting the drug to influences such as gastric acid, intestinal enzymes, and other variables. Moreover, oral administration entails a first-pass effect, wherein a fraction of the drug undergoes hepatic metabolism, leading to comparatively diminished blood concentrations postadministration (Kwan, 1997). Therefore, different routes of administration may result in varying blood concentrations of VRC, even when the agent is administered at the dosage recommended in the prescribing information.

In addition, VRC undergoes extensive hepatic metabolism via the cytochrome P450 system (mainly *CYP2C19, CYP3A4* and *CYP2C9*) (Schulz et al., 2019). The variability of VRC exposure is related to the presence of *CYP2C19* polymorphisms, and the most common defective alleles are CYP2C19*2, CYP2C19*3, CYP2C19*4, and CYP2C19*17 (Dean et al., 2012). Dosing based on the *CYP2C19* genotype have been recommended at the highest level by the Clinical Pharmacogenetics Implementation Consortium (CPIC)(Level A recommendation) and PHARMGKB(Level 1A recommendation) (Dean et al., 2019).^1^ However, there is limited research on the impact of CYP2C19 gene polymorphisms on VRC concentrations in Chinese patients with hematologic malignancies under different administration routes. Furthermore, the influence of CYP3A4 genotype and ABCB1 transporter protein on VRC concentrations remains controversial (Fan et al., 2022; Liu et al., 2024; Chuwongwattana et al., 2020), and a low level of evidence (level 3 recommendation) is provided by PHARMGKB.^1^ Thus, further confirmation is needed to determine whether genetic variations in CYP3A4 and ABCB1 affect the pharmacokinetics of VRC.

Therefore, the current study aimed to examine the influence of different administration routes and gene polymorphisms of *CYP2C19*, *CYP3A4* and *ABCB1* on the serum VRC concentration among Chinese patients with hematologic malignancies.

## 2 Materials and methods

### 2.1 Patients, data collection and blood collection

Patients who were receiving VRC for prophylaxis/treatment of IFDs were enrolled from March 2017 to February 2018 at the First Affiliated Hospital of Soochow University, China. IFDs were defined and classified according to the definitions of the Invasive Fungal Infection Group of the European Organization for Research and Treatment of Cancer and Mycoses Study Group of the National Institute of Allergy and Infectious Diseases. Follow-up visit data were updated via telephone, and medical records were reviewed. The data used for research purposes were approved by the Institutional Research Ethics Committee. The exclusion criteria were as follows: 1) sampling was obtained prior to reaching a steady-state trough concentration that was defined as a level obtained after 3 days of therapy with VRC, and the samples were collected at intervals of 10–12 h post-administration; 2) initial TDM occurred after dose adjustment; or 3) missing data.

Venous blood was collected by EDTA anticoagulation tubes no earlier than before the fifth dose, i.e., 30 min before dosing on the third day, at the loading dose; without the loading dose drug concentrations reached steady state on the seventh day, and venous blood was collected 30 min before dosing.

**FIGURE 1:**
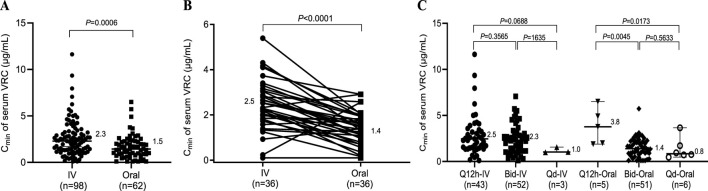
Effects of different administration routes on the serum concentration of VRC. **(A)** Serum concentrations of VRC obtained by different routes of administration; **(B)** Serum concentrations of VRC obtained from the same patients who received both intravenous and oral administration. **(C)** Serum concentrations of VRC obtained at different dosing intervals. Q12h, dosing every 12 h; Bid, dosing twice a day; Qd, dosing once a day; IV, intravenous.

A total of 160 patients with hematological malignancies were retrospectively enrolled in our study. Among the 160 enrolled patients, 62 patients were administered orally and 98 patients were administered intravenously. The first point of venous blood was collected for each 160 patients. Among the 98 patients who received intravenous administration, 36 patients later switched to oral administration for certain reasons. We collected venous blood samples from these 36 patients after intravenous and oral administration respectively. For these 36 patients who changed from intravenous to oral administration, the VRC concentrations of their intravenous infusion were included in the 98 cases of intravenous patients for subsequent result analysis, the VRC concentrations results of their oral administration were only utilized for the analysis of Figure 1B and were no longer employed for the statistical analysis of the subsequent results.

### 2.2 Determination of the VRC concentration

The serum was separated from each subject through a centrifugation process (3500 × g, 10 min). The measurement of the serum VRC concentrations was performed at the study center via the methods described in our previous publication (Wang et al., 2017). In brief, VRC (purity: 99.9%) was provided by Chengdu Huashen Group Co. (Chengdu, China). Fentanyl (internal standard, IS, purity: 100%) was supplied by Yichang Renfu Pharmaceutical Co. (Yichang, China). The UPLC-MS/MS system was composed of a 6410 tandem mass spectrometer and 1200 liquid chromatography system, which were both from Agilent Technologies Ltd. Chromatographic conditions: (1) Chromatographic column: XDBC18 (4.6 mm × 50 mm, 1.8 µm), mobile phase: acetonitrile-10 mmol/L ammonium formate (pH = 3.05) (55∶45), flow rate: 1.0 min/mL, and column temperature: 30°C. (2) Mass spectrometry conditions: capillary voltage of 4500 V, drying gas flow rate of 6 min/L, atomizing gas pressure of 413.7 kPa, drying gas temperature of 350°C; VRC: fragmentation voltage of 100 V, collision energy of 15 V, ion pair: m/z 350.2/281.2; fentanyl: fragmentation voltage of 135 V, collision energy of 25 V, ion pair: m/z 350.2/281.2; fentanyl: fragmentation voltage of 135 V, collision energy of 25 V, ion pair: m/z 350.2/281.2 Fentanyl: Fragmentation voltage of 135 V, collision energy of 25 V, ion pair: m/z 337.3/188.2.

### 2.3 Genotyping

Two milliliters of whole blood from each subject was collected into EDTA-Vacutainer tubes. DNA was purified via the Magen HiPure BloodDNA Mini Kit method. The non-functional CYP2C192 allele (rs4244285) and CYP2C193 (rs4986893) were detected. Additionally, the increased function CYP2C19*17 allele (rs12248560) was also identified. The rs4646437, rs2242480, and rs2246709 in the intronic region of CYP3A4 were detected in accordance with the references (Fan et al., 2022; Liu et al., 2024). Moreover, the three most common SNPs (rs1128503(Gly412Gly), rs2032582(Ser893Ala/Thr), rs1045642(le1145lle)) in the protein coding region of ABCB1 were detected. Nine SNPs were genotyped via multiplex PCR and sequencing (Sangon, Shanghai, China). The sequence of all primers were showed in Table 1. A panel that contains 10 target SNP sites was designed. Library preparation was performed via two-step PCR. The first round of PCR was performed as follows: 2 μL of DNA (10 ng/μL), 1 μL of amplicon PCR forward primer mixture (10 μM), 1 μL of amplicon PCR reverse primer mixture (10 μM), and 15 μL of 2×PCR Ready Mix (total 25 μL) (Kapa HiFi Ready Mix). The plate was sealed, and PCR was performed in a thermal instrument (Bio-Rad, T100TM) via the following program: 1 cycle of denaturation at 98°C for 5 min; 8 cycles of denaturation at 98°C for 30 s, annealing at 50°C for 30 s, and elongation at 72°C for 30 s; and 25 cycles of denaturation at 98°C for 30 s, annealing at 66°C for 30 s, elongation at 72°C for 30 s and a final extension at 72°C for 5 min. Finally, the samples were incubated at 4°C. The PCR products were checked via electrophoresis in 1% (w/v) agarose gels in TBE buffer (Tris, boric acid, EDTA) stained with ethidium bromide (EB) and visualized under UV light. We subsequently used AMPure XP beads to purify the amplicon product. Afterward, a second round of PCR was performed. The PCR mixtures used were as follows: 2 μL of DNA (10 ng/μL), 1 μL of universal P7 primer with a barcode (10 μM), 1 μL of universal P5 primer (10 μM), and 15 μL of 2× PCR Ready Mix (for a total of 30 μL) (Kapa HiFi Ready Mix). The plate was sealed, and PCR was performed in a thermal instrument (Bio-Rad, T100TM) using the following program: 1 cycle of denaturation at 98°C for 3 min, then 5 cycles of denaturation at 94°C for 30 s, annealing at 55°C for 20 s, elongation at 72°C for 30 s, and a final extension at 72°C for 5 min. Then, we used AMPure XP beads to purify the amplicon product. The libraries were then quantified and pooled. Paired-end sequencing of the library was performed on HiSeq X Ten sequencers (Illumina, San Diego, CA).

**TABLE 1:** Sequence of PCR primers.

Gene name	rs ID	Foward	Reverse
CYP2C19	rs4244285	ATC​AAT​AAA​GTC​CCG​AGG​GTT​GTT​G	ATT​ACA​ACC​AGA​GCT​TGG​CAT​ATT​G
CYP2C19	rs4986893	AAT​GTA​CTT​CAG​GGC​TTG​GTC​AAT​A	GTT​TCC​AAT​CAT​TTA​GCT​TCA​CCC​T
CYP2C19	rs12248560	ATC​GTG​GCG​CAT​TAT​CTC​TTA​CAT​C	CTG​TTT​TCC​TTA​GAT​AAA​TAA​GTG​G
CYP3A4	rs4646437	AGC​AAG​ATT​AAT​TTT​GAG​CTT​CAG​A	CCA​ACC​AGA​AGA​GTA​AAA​GAC​ATC​A
CYP3A4	rs2242480	AGA​AAC​TGC​AGG​AGG​AAA​TTG​ATG​C	TAA​TAG​AAA​GCA​GAT​GAA​CCA​GAG​C
CYP3A4	rs2246709	ACC​TCA​TAC​ATT​TTT​AGC​TAT​CAG​C	AAA​TCA​GTA​ATC​TAT​GTT​CAT​GCC​A
ABCB1	rs1128503	GAA​CAG​TCA​GTT​CCT​ATA​TCC​TGT​G	TTG​AAA​GGG​CAA​CAT​CAG​AAA​GAT​G
ABCB1	rs2032582	TCC​TTC​ATC​TAT​GGT​TGG​CAA​CTA​A	ATG​AAA​AAG​ATT​GCT​TTG​AGG​AAT​G
ABCB1	rs1045642	CTG​GTC​CTG​AAG​TTG​ATC​TGT​GAA​C	TCC​CAG​GCT​GTT​TAT​TTG​AAG​AGA​G

After the sequencing step, raw reads were filtered according to two steps: (1)Removing adaptor sequence if reads contains by cutadapt (v 1.2.1); (2) Removing low quality bases from reads 3′ to 5′ (Q < 20) by PRINSEQ-lite(v 0.20.3); And the remaining clean data were mapped to the reference genome by BWA(version 0.7.13-r1126) with default parameters. Samtools (Version: 0.1.18) was used to calculate each genotype of target site. Annovar (2018-04-16) was used to detect genetic variants.

The metabolic phenotype of each patient was identified as follows: ultrarapid metabolizer (UM) [CYP2C19*17/*17], rapid metabolizer (RM) [CYP2C19*1/*17], extensive metabolizer (EM) [CYP2C19*1/*1], intermediate metabolizer (IM) [CYP2C19*1/*2 and CYP2C19*1/*3], and poor metabolizer (PM) [CYP2C19*2/*2, CYP2C19*2/*3, and CYP2C19*3/*3].

### 2.4 Statistical analysis

Data processing and analysis were conducted using SPSS 26.0 statistical software. The nonparametric Mann-Whitney U test and Kruskal-Wallis test were used to compare continuous variables. A p-value of <0.05 was considered statistically significant.

## 3 Results

### 3.1 Patient characteristics

A total of 160 patients with hematological malignancies were retrospectively enrolled in our study. Among the 160 patients, 96 (60.0%) were male, and 64 (40.0%) were female. The mean age of the patients was 37.13 ± 15.70 years. The op three most common underlying diseases were acute myelogenous leukemia (61, 38.1%), acute lymphocytic leukemia (27, 16.9%), and myelodysplastic syndrome (22, 13.8%). Among the 160 serum samples, 62 (38.7%) were oral, and 98 (61.3%) were intravenous. Among the oral samples, patients received 0.2 g VRC twice a day (Bid) or once every 12 h (Q12h). Among the intravenous samples, approximately 98% (95/98) are administered in Q12h and Bid. For patients administered in Q12h and Bid, around 78% (77/95) have a maintenance dose of VRC ranging from 3.5 to 4.5 mg/kg. The patient demographics and characteristics in this study are summarized in Table 2.

**TABLE 2:** Characteristics of 160 patients enrolled in this study.

Characteristic	N (%) or median (IQR)
Demographics of patients	160
Age (years)	37.5 (9-88)
Sex, Male/Female	96/64
Underlying condition	
Acute myelogenous leukemia	61 (38.1%)
Acute lymphocytic leukemia	27 (16.9%)
Myelodysplastic syndrome	22 (13.8%)
Other	50 (31.2%)
VRC form during sampling	
Oral	62(38.7%)
Intravenous	98(61.3%)
VRC dose during sampling	
Oral form only	0.2 g Bid/Q12h
Intravenous form (N = 98)	3.92 mg/kg (2.47 mg/kg −5.88 mg/kg)
Intravenous form (Bid, N = 52)	4.16 mg/kg (2.78 mg/kg −5.88 mg/kg)
Intravenous form (Q12h, N = 43)	3.89 mg/kg (2.47 mg/kg −5.56 mg/kg)

### 3.2 Effects of different administration routes on the serum C_min_ of VRC

In clinical practice, VRC is mainly administered intravenously or orally. Therefore, we first investigated the influence of the administration route and dosing interval on the C_min_ of VRC. As shown in Figure 1A, a significant difference was observed between intravenous and oral administration (2.3 vs. 1.5 μg/mL, *P* = 0.0006), and the C_min_ of intravenous VRC was 1.5 times greater than the C_min_ of oral VRC at the dosage recommended in the prescribing information. To further clarify the difference between intravenous and oral administration, the C_min_ of VRC from the same patients who received both intravenous and oral administration were detected. A significant difference was still observed between intravenous and oral administration (2.5 vs. 1.4 μg/mL, *P* < 0.0001) to the same patients (Figure 1B). Moreover, there was no difference in the C_min_ of the serum VRC between Q12h and Bid after intravenous administration. However, the C_min_ of serum VRC appears to be greater in those taking VRC by Q12h than in those receiving Bid or Qd via oral administration (3.8 vs. 1.4 μg/mL, *P* = 0.0045; 3.8 vs. 0.8 μg/mL, *P* = 0.0173).

### 3.3 The effect of the *CYP2C19* genotype on the C_min_ of serum intravenous and oral VRC

Considering the practical clinical scenario, the assessment of patients’ genetic polymorphisms typically does not account for dosing intervals. Therefore, in the following analysis, the IV + Oral group included all patients, representing the general clinical setting. As indicated in Section 3.2, dosing intervals in the IV group do not impact VRC concentrations. Hence, all patients in the IV group were included. Given that dosing intervals affect VRC concentrations in the Oral group, only patients who were administered VRC by Bid were included.

**FIGURE 2:**
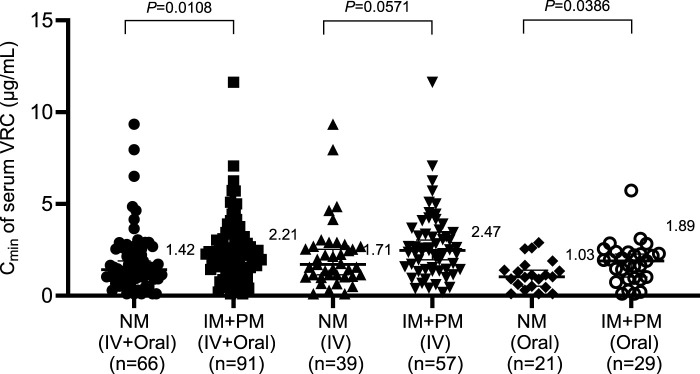
Effects of the *CYP2C19* genotype on the serum concentration of VRC. IV, intravenous.

According to the genotyping results of *CYP2C19* among 160 patients receiving VRC, 66 patients were normal metabolizers (NMs), 77 patients were intermediate metabolizers (IMs), and 14 patients were poor metabolizers (PMs). Because only 3 patients were rapid metabolizers (RMs) and no ultrarapid metabolizers (UMs) were included, patients with rapid metabolizers and ultrarapid metabolizers were excluded because of the limited sample size. The effects of the *CYP2C19* genotype on the serum concentrations of intravenous and oral VRC were subsequently analyzed, and the results are shown in Figure 2. Within the IV + Oral and Oral groups, the C_min_ of the serum VRC in the NMs was significantly lower than that in the IM + PM group (1.42 vs. 2.21, *P* = 0.0108; 1.03 vs. 1.89, *P* = 0.0386). Although no statistically significant difference was observed within the IV group, the C_min_ of the serum VRC in the NMs was also lower than that in the IM + PM group (1.71 vs. 2.47, *P* = 0.0571).

### 3.4 Effects of the *CYP3A4* genotype on the C_min_ of the serum intravenous and oral VRC

**FIGURE 3:**
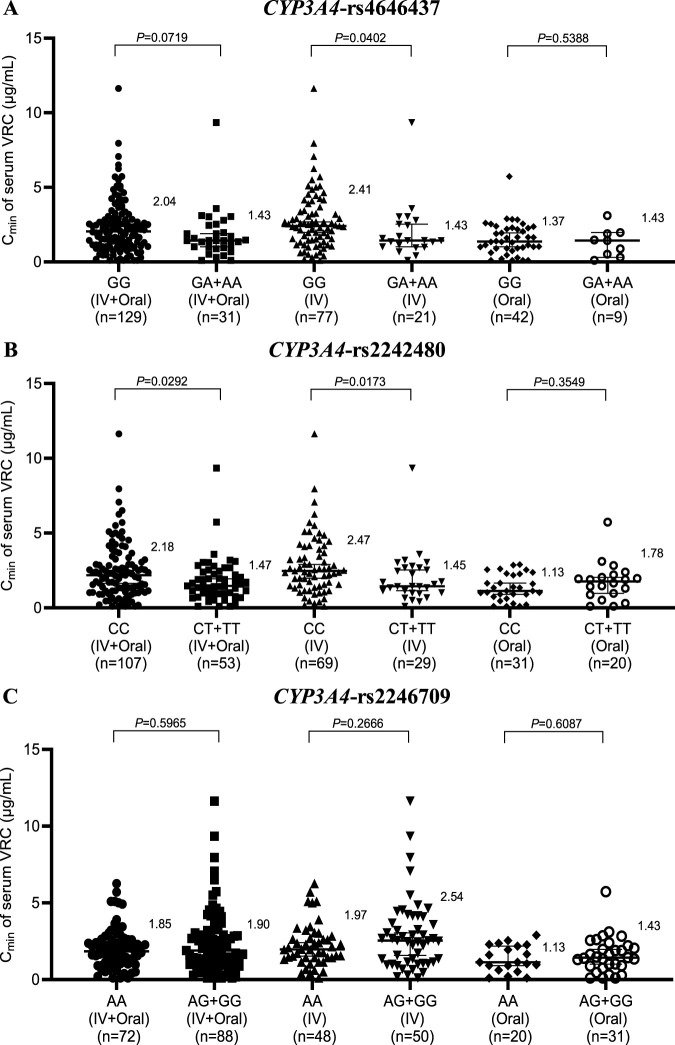
Effects of the *CYP3A4* genotype on the serum concentration of VRC in the rs4646437 group **(A)**, rs2242480 group **(B)** and rs2246709 group **(C)**. IV, intravenous.

The genotyping results for rs4646437 (GG, GA + AA), rs2242480 (CC, CT + TT), and rs2246709 (AA, AG + GG) for *CYP3A4* in 160 patients are shown in Figure 3. Patients with the wild-type *CYP3A4* rs4646437 genotype had significantly higher VRC concentrations than those with the mutant genotype within the IV group (2.41 vs. 1.43, *P* = 0.0402), but no significant difference was observed within the IV + Oral or Oral group (Figure 3A). On the other hand, patients with the wild-type *CYP3A4* rs2242480 genotype presented markedly elevated VRC concentrations compared with those with the mutant genotype within the (IV + Oral) and IV groups (2.18 vs. 1.47, *P* = 0.0292; 2.47 vs. 1.45, *P* = 0.0173) (Figure 3B), but no significant difference was observed in the Oral group. Furthermore, no significant difference was detected in any of the rs2246709 groups (Figure 3C).

### 3.5 Effects of *ABCB1* genotype on the C_min_ of serum intravenous and oral VRC

**FIGURE 4:**
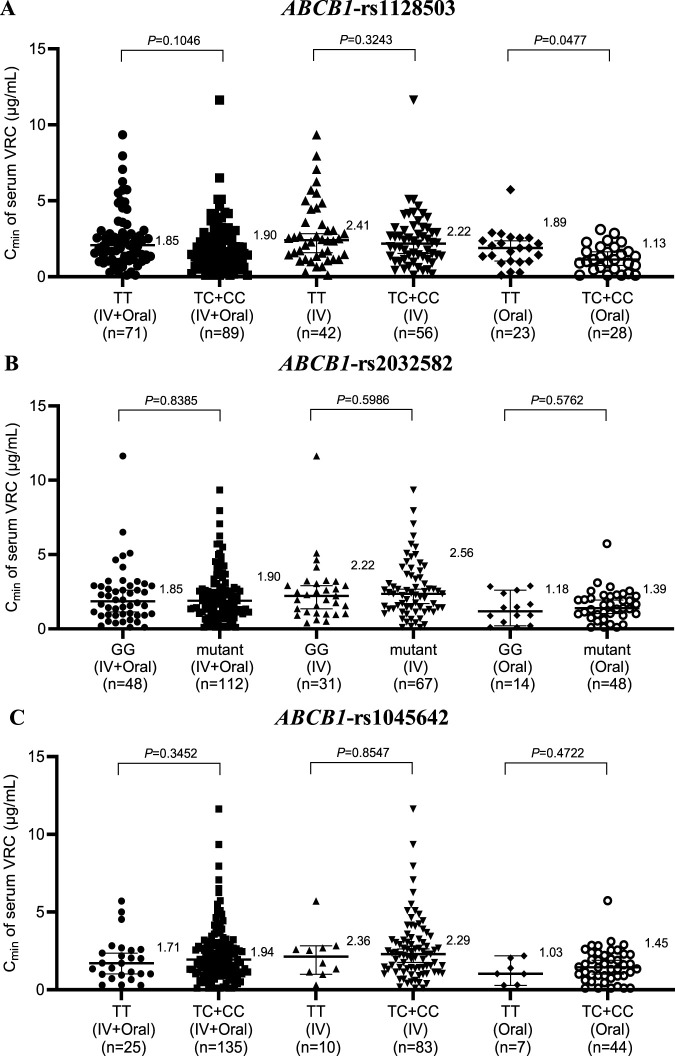
Effects of *ABCB1* genotype on the serum concentration of VRC in the rs1128503 group **(A)**, rs2032582 group **(B)** and rs1045642 group **(C)**. IV, intravenous.

The three most common SNPs of *ABCB1* in the protein coding region are rs1128503, rs2032582 and rs1045642. These three SNPs have been the focus of many pharmacokinetic and disease association studies with controversial results. Thus, genotyping results based on rs1128503, rs2032582, and rs1045642 for *ABCB1* in 160 patients are shown in Figure 4. Within the Oral group, patients with the wild-type *ABCB1* rs1128503 genotype presented notably higher VRC concentrations than those with the mutant genotype (1.89 vs. 1.13, *P* = 0.0477) (Figure 4A). However, no significant difference was observed within the IV + Oral or IV group of rs1128503 or any groups of rs2032582 (Figure 4B) or rs1045642 (Figure 4.C).

## 4 Discussion

VRC is a first-line drug for the treatment of IFDs that exhibits nonlinear pharmacokinetic properties, and its serum concentration varies widely between and within individuals (Zhong et al., 2018). Therefore, it is essential to identify potential factors that contribute to variations in VRC concentrations to develop individualized VRC therapy. However, the limited data on Chinese patients with hematologic malignancies make it difficult to make dosage decisions for such patients in clinical practice (Malani et al., 2015). Therefore, the aim of this study was to examine the effects of the route of administration and genetic polymorphisms of *CYP2C19*, *CYP3A4*, and *ABCB1* on the serum concentrations of VRC.

First, the C_min_ of intravenous VRC was greater than the C_min_ of oral VRC when patients were treated at the dosage recommended in the prescribing information, which is consistent with findings from previous studies (Harada et al., 2021). The difference between administration routes may be due to the absence of an absorption process for intravenous administration. Additionally, when administered orally, patients taking VRC by Q12h seem to have a higher C_min_ of serum VRC than those receiving VRC by Bid and Qd. This difference may be attributed to the fact that strict dosing intervals favor higher VRC concentrations in patients.

Second, the influence of the *CYP2C19* genotype on VRC concentration significantly differed between the IV + Oral and Oral groups, which is consistent with findings from previous studies (Anonymous, 2018). Although no statistically significant difference was observed in the IV group, there was a general trend of increasing C_min_ of the serum VRC between the NM and (IM + PM) groups, and the *P* value was close to 0.05, which is consistent with previous reports (Dean et al., 2019).

Furthermore, in both the IV + Oral and IV groups of *CYP3A4* rs2242480, the C_min_ of serum VRC in the CCs was markedly greater than that in the CT + TT group; however, no significant difference was noted in the Oral group. Similarly, within the IV group of *CYP3A4* rs4646437, the C_min_ of the serum VRC in the GGs was significantly greater than that in the GA + AA group; however, no notable difference was observed in either the IV + Oral or Oral groups, which is consistent with the findings from Su-jie Jia’s study (Jia et al., 2021). No significant differences were detected across the groups for *CYP3A4* rs2246709. Moreover, in the Oral group for *ABCB1* rs1128503, patients with the wild-type genotype presented significantly greater serum VRC C_min_ values than those with the mutant genotype. However, no substantial disparity was noted within the IV + Oral or IV group of rs1128503 or across any groups of rs2032582 or rs1045642.

Based on the aforementioned results, all gene polymorphisms, except *CYP2C19*, *CYP3A4* rs2242480, *CYP3A4* rs4646437, and *ABCB1* rs1128503, exhibit minimal effects on the *in vivo* serum C_min_ of VRC. This observation aligns with the findings of Beibei Shao (Wang et al., 2017). In clinical practice, genotype testing typically does not consider dosage forms. These findings suggest that the *CYP3A4* rs2242480 and *CYP3A4* rs4646437 genotypes primarily affect VRC concentrations during intravenous administration, thus rendering genotype testing less crucial during oral administration. In contrast, the effect of the *ABCB1* rs1128503 genotype was the opposite.

Despite our investigation into administration route and gene polymorphisms on the serum concentration of VRC, there are still several areas for improvement in our article: 1) Due to the extremely low mutation frequency of certain SNP sites (for instance, the mutation frequency of CYP2C19*17 is 1.9% (3/160), some phenotypes had relatively small sample sizes (only 3 patients were rapid metabolizers (RMs) and there were no ultrarapid metabolizers (UMs)). This may have an impact on the statistical significance and the reliability of the conclusions. Subsequently, it was necessary to enlarge the sample size to validate the finding. 2) In addition to administration routes and genes, gender, age, liver function status, and concomitant medication can all have an impact on VRC concentrations (Harada et al., 2021; Allegra et al., 2020; You et al., 2018). In future studies, multivariate analysis or population pharmacokinetic models are preferable for the comprehensive analysis of multiple influencing factors. 3) The relationship between the efficacy/safety of VRC and the C_min_ of VRC under different administration routes and different genotypes requires further investigation.

In conclusion, the C_min_ of intravenous VRC is greater than that of oral VRC when administered at the recommended dosage. It is optimal for patients to take VRC by Q12h when it is administered orally in order to obtain a higher C_min_ of serum VRC. Additionally, attention should be devoted to VRC serum concentrations in patients with mutations in *CYP2C19*. The *CYP3A4* rs2242480 and *CYP3A4* rs4646437 genotypes may primarily affect VRC concentrations during intravenous administration, whereas *ABCB1* rs1128503 primarily affects VRC concentrations during oral administration.

## Data Availability

The original contributions presented in the study are publicly available. This data can be found here: https://www.ncbi.nlm.nih.gov/bioproject/?term=PRJNA1279949.

## Footnotes

1
https://www.pharmgkb.org

## References

[B1] Anonymous (2018). Clinical Pharmacogenetics implementation Consortium (CPIC) guidelines for CYP2C19 and voriconazole therapy. Clin. Pharmacol. Ther. 103 (2), 349. 10.1002/cpt.953 29313967

[B2] Allegra S. De Francia S. De Nicolo A. Cusato J. Avataneo V. Manca A. (2020). Effect of gender and age on voriconazole trough concentrations in Italian adult patients. Eur. J. drug metabolism Pharmacokinet. 45 (3), 405–412. 10.1007/s13318-019-00603-6 31965553

[B3] Bassetti M. Azoulay E. Kullberg B. J. Ruhnke M. Shoham S. Vazquez J. (2021). EORTC/MSGERC definitions of invasive fungal diseases: summary of activities of the intensive care unit working group. Clin. Infect. Dis. official Publ. Infect. Dis. Soc. Am. 72 (Suppl. 2), S121–S127. 10.1093/cid/ciaa1751 33709127

[B4] Boglione-Kerrien C. Zerrouki S. Le Bot A. Camus C. Marchand T. Bellissant E. (2023). Can we predict the influence of inflammation on voriconazole exposure? An overview. J. Antimicrob. Chemother. 78 (11), 2630–2636. 10.1093/jac/dkad293 37796931

[B5] Chuwongwattana S. Jantararoungtong T. Prommas S. Medhasi S. Puangpetch A. Sukasem C. (2020). Impact of CYP2C19, CYP3A4, ABCB1, and FMO3 genotypes on plasma voriconazole in Thai patients with invasive fungal infections. Pharmacol. Res. & Perspect. 8 (6), e00665. 10.1002/prp2.665 33124772 PMC7596670

[B6] Dean L. (2012). “Voriconazole therapy and CYP2C19 genotype,” in *Medical genetics summaries*: bethesda (MD) Editors Pratt V. M. Scott S. A. Pirmohamed M. Esquivel B. Kattman B. L. Malheiro A. J. (Bethesda MD: National Center for Biotechnology Information US). 31886997

[B7] Dean L. (2019). “Voriconazole therapy and CYP2C19 genotype,” in *Medical genetics summaries*: bethesda (MD) Editors Pratt V. M. Scott S. A. Pirmohamed M. Esquivel B. Kattman B. L. Malheiro A. J. (Bethesda MD: National Center for Biotechnology Information US). 31886997

[B8] Fan X. Zhang H. Wen Z. Zheng X. Yang Y. Yang J. (2022). Effects of CYP2C19, CYP2C9 and CYP3A4 gene polymorphisms on plasma voriconazole levels in Chinese pediatric patients. Pharmacogenetics genomics 32 (4), 152–158. 10.1097/FPC.0000000000000464 35081606

[B9] Godoy M. C. B. Ferreira D. P. H. R. Truong M. T. Shroff G. S. Marom E. M. (2022). Invasive fungal pneumonia in immunocompromised patients. Radiologic Clin. N. Am. 60 (3), 497–506. 10.1016/j.rcl.2022.01.006 35534133

[B10] Harada S. Niwa T. Hoshino Y. Fujibayashi A. Suzuki A. (2021). Influence of switching from intravenous to oral administration on serum voriconazole concentration. J. Clin. Pharm. Ther. 46 (3), 780–785. 10.1111/jcpt.13352 33393135

[B11] Hoenigl M. Duettmann W. Raggam R. B. Seeber K. Troppan K. Fruhwald S. (2013). Potential factors for inadequate voriconazole plasma concentrations in intensive care unit patients and patients with hematological malignancies. Antimicrob. agents Chemother. 57 (7), 3262–3267. 10.1128/AAC.00251-13 23629724 PMC3697337

[B12] Jia S. J. Gao K. Q. Huang P. H. Guo R. Zuo X. C. Xia Q. (2021). Interactive effects of glucocorticoids and cytochrome P450 polymorphisms on the plasma trough concentrations of voriconazole. Front. Pharmacol. 12, 666296. 10.3389/fphar.2021.666296 34113252 PMC8185288

[B13] Kwan K. C. (1997). Oral bioavailability and first-pass effects. Drug metabolism Dispos. Biol. fate Chem. 25 (12), 1329–1336. 9394021

[B14] Liu S. Yao X. Tao J. Zhao S. Sun S. Wang S. (2024). Impact of CYP2C19, CYP2C9, CYP3A4, and FMO3 genetic polymorphisms and sex on the pharmacokinetics of voriconazole after single and multiple doses in healthy Chinese subjects. J. Clin. Pharmacol. 64 (8), 1030–1043. 10.1002/jcph.2440 38654529

[B15] Malani A. N. Kerr L. E. Kauffman C. A. (2015). Voriconazole: how to use this antifungal agent and what to expect. Seminars Respir. Crit. care Med. 36 (5), 786–795. 10.1055/s-0035-1562903 26398543

[B16] Schulz J. Kluwe F. Mikus G. Michelet R. Kloft C. (2019). Novel insights into the complex pharmacokinetics of voriconazole: a review of its metabolism. Drug metab. Rev. 51 (3), 247–265. 10.1080/03602532.2019.1632888 31215810

[B17] Stemler J. Mellinghoff S. C. Khodamoradi Y. Sprute R. Classen A. Y. Zapke S. E. (2023). Primary prophylaxis of invasive fungal diseases in patients with haematological malignancies: 2022 update of the recommendations of the Infectious Diseases Working Party (AGIHO) of the German Society for Haematology and Medical Oncology (DGHO). J. Antimicrob. Chemother. 78 (8), 1813–1826. 10.1093/jac/dkad143 37311136 PMC10393896

[B18] Theuretzbacher U. Ihle F. Derendorf H. (2006). Pharmacokinetic/pharmacodynamic profile of voriconazole. Clin. Pharmacokinet. 45 (7), 649–663. 10.2165/00003088-200645070-00002 16802848

[B19] Waitt C. Waitt P. Pirmohamed M. (2004). Intravenous therapy. Postgrad. Med. J. 80 (939), 1–6. 10.1136/pmj.2003.010421 14760169 PMC1757963

[B20] You H. Dong Y. Zou Y. Zhang T. Lei J. Chen L. (2018). Voriconazole therapeutic drug monitoring: factors associated with supratherapeutic and subtherapeutic voriconazole concentrations. Int. J. Clin. Pharmacol. Ther. 56 (5), 239–246. 10.5414/CP203184 29393850

[B21] Zhong X. Tong X. Ju Y. Du X. Li Y. (2018). Interpersonal factors in the pharmacokinetics and pharmacodynamics of voriconazole: are CYP2C19 genotypes enough for us to make a clinical decision? Curr. drug Metab. 19 (14), 1152–1158. 10.2174/1389200219666171227200547 29361899 PMC6635675

[B22] Wang T. Li C. Gu C. Chen X. Dong J. Yang D. (2017). Therapeutic drug monitoring for voriconazole in patients with invasive fungal infections. Chin Hosp Pharm J. 37 (21), 2151–2154. 10.13286/j.cnki.chinhosppharmacyj.2017.21.11

